# Enhancement of Kinematic Accelerations by Wavenumber Correlation Filtering

**DOI:** 10.3390/s16091434

**Published:** 2016-09-06

**Authors:** Chang-Ki Hong, Jay Hyoun Kwon

**Affiliations:** 1 Department of Geoinformatics Engineering, Kyungil University, Daegu 38428, Korea; ckhong@kiu.ac.kr; 2 Department of Geoinformatics, University of Seoul, Seoul 02504, Korea

**Keywords:** GPS, kinematic acceleration, wavenumber correlation filter, airborne gravimetry

## Abstract

To obtain kinematic accelerations with high accuracy and reliability, multiple Global Positioning System (GPS) receivers with a single antenna can be used for airborne gravimetry. The data collected from each receiver can be processed for kinematic accelerations that may be combined using simple averaging. Here, however, uncorrelated errors from instrument errors in each receiver also will be included that degrade the final solutions. Therefore, in this study, the wavenumber correlation filter (WCF) is applied to extract only the higher positively correlated wavenumber components of the kinematic accelerations for the enhancement of the final solution. The in situ airborne GPS data from two receivers were wavenumber-correlation-filtered to show about 0.07835 Gal improvement in accuracy relative to the solution from the raw kinematic accelerations.

## 1. Introduction

Global Positioning System (GPS) has been widely used in the fields which require accurate positioning in 3-D space. Nowadays, however, the GPS is also used in various non-positioning applications such as GPS meteorology and geodesy [1,2,3]. One of the GPS applications in geodesy provides kinematic accelerations for airborne gravity surveying. The measured kinematic accelerations of the aircraft $\ddot{x}$ are related to the earth’s gravitational acceleration g and other accelerations a according to the well-known navigation equation [4] given by

(1) $$\ddot{x}=g+a$$

By Equation (1), the accuracy of the gravity depends directly on the accuracy of the measured kinematic accelerations. The kinematic accelerations, in turn, are usually computed by taking second-order time-derivatives of the aircraft’s positions measured in relative positioning mode using single or multiple GPS stations [5]. The kinematic accelerations can be estimated directly using the position-velocity-acceleration (PVA) model [6,7], which does not require taking the additional time-derivatives of the positions. However, both methods basically acquire precise kinematic acceleration information using relative GPS positioning that involves multiple receivers with a single antenna installed on the aircraft. Moreover, the multiple GPS receivers facilitate separating the meaningful signals from the receiver’s errors because the receivers share the single antenna’s signals.

In general, the gravity-based kinematic accelerations from multiple receivers should exhibit high positively correlated signals, whereas the minimally or negatively correlated signals reflect measurement noise in the kinematic accelerations. Thus, to separate the highly correlated signals from the noise components, the wavenumber correlation filter (WCF) can be applied, which computes correlation coefficients (CCs) between the signal’s frequency components according to the cosines of their phase differences. This method has been successfully applied to the geophysical and geodetic data obtained from the repeated survey tracks to significantly improve their signal-to-noise properties [5,8,9,10,11,12] approximated by

(2) $$n/s\;∼\sqrt{1/\left|CC\right|-1}$$

In this paper, airborne kinematic accelerations are enhanced using the WCF. The signal-to-noise results show significant accuracy improvement and can be obtained by suppressing the minimally and negatively correlated wavenumber components. 

## 2. Methodology

**Figure 1:**
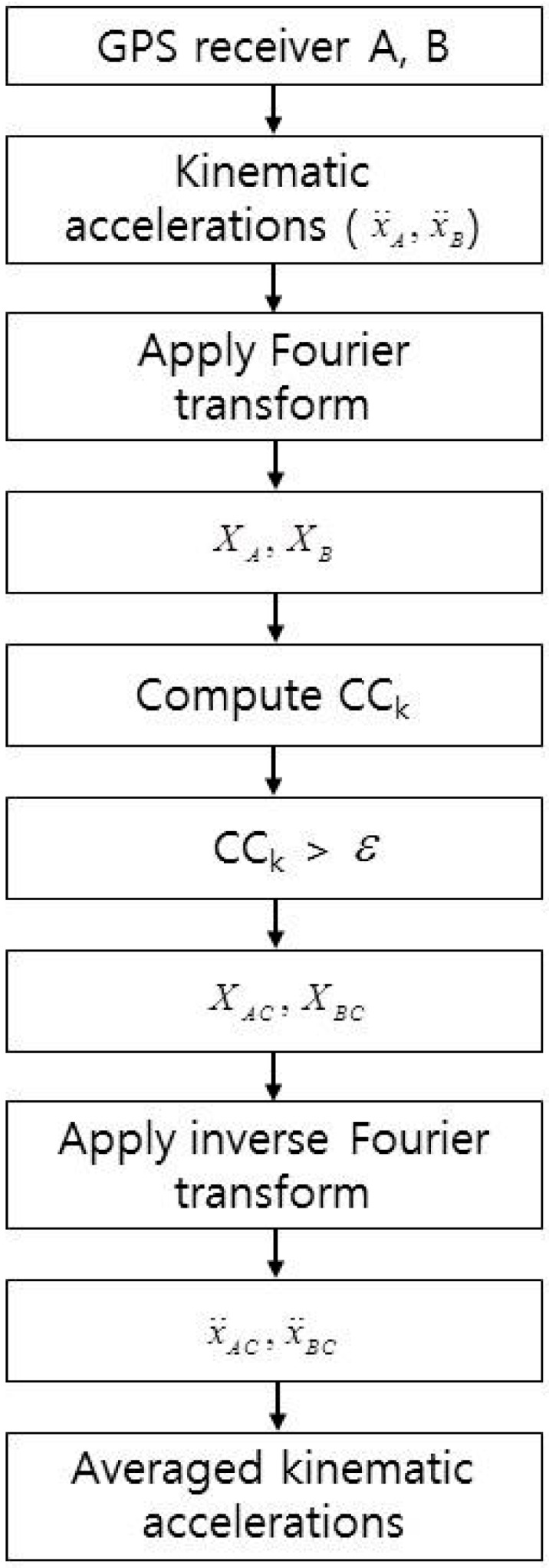
Flowchart of data processing.

Figure 1 shows the overall methodology adopted in this study. GPS receivers A and B installed on an aircraft simultaneously collect GPS measurements. The kinematic accelerations of the aircraft are computed using a network-based kinematic positioning technique with the PVA model which avoids numerical differentiation of the positions to obtain kinematic accelerations. The PVA model includes only the positions, but also the velocities and kinematic accelerations, of the aircraft in the Kalman filter’s state. Also, the corresponding transition matrix, which describes the dynamic of aircraft and GPS measurement model, is constructed for medium- to long-range kinematic applications. More details on the PVA model and the approaches adopted in this study can be found in [6].

To apply WCF, the Fourier transforms are taken of the estimated kinematic accelerations from both receivers. The kinematic accelerations are next analyzed for their *k*-th wavenumber correlation coefficients $CC_{k}$ using

(3) $$CC_{k}=\frac{X_{A,k}\cdot X_{B,k}}{\left|X_{A,k}\right|\left|X_{B,k}\right|}$$

where the numerator is the dot product of the *k*-th Fourier transform wavevectors of the kinematic accelerations for receiver A and B, and the denominator is the cross-power of the wavevectors [8,9].

The CC for each frequency component from both datasets can be computed and compared with the predefined correlation tolerance ε. If the computed CC is larger than the tolerance, then that frequency component is passed as signal. Otherwise, it is assumed to be noise and rejected. In this study, the correlation tolerance was set to 0.9 because very high correlations are expected. Next, the WCF kinematic accelerations are recovered by inversely transforming the wavenumber components that exceed the preset correlation tolerance. Finally, the kinematic accelerations are least squares estimated by averaging the WCF kinematic accelerations. Evaluations of signal improvement are performed using the root-mean-squared (RMS) differences in the unfiltered and filtered receiver accelerations and their related signal-to-noise properties (Equation (2)).

## 3. Numerical Results

**Figure 2:**
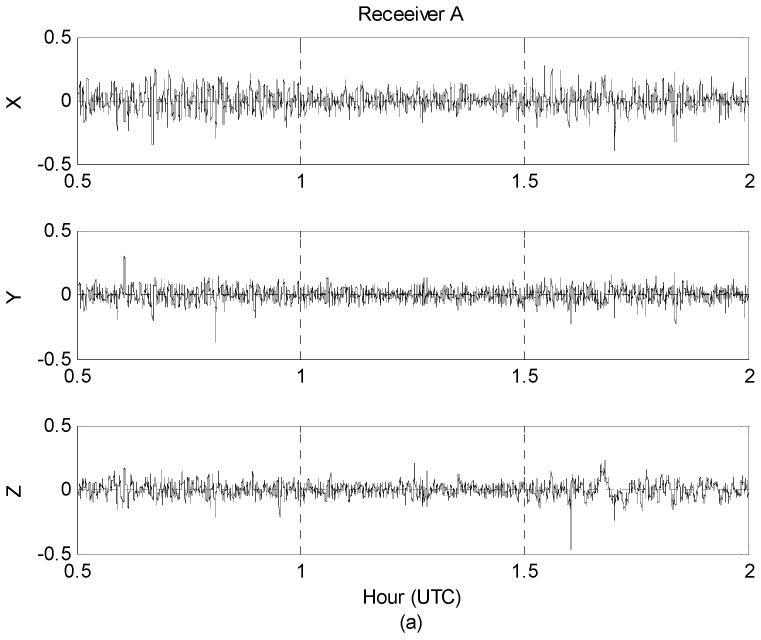

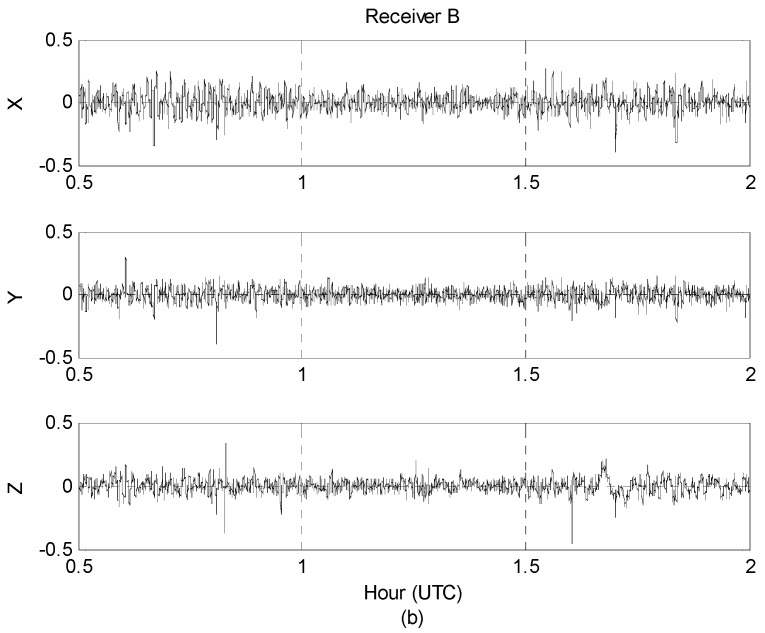
Estimated three-component kinematic accelerations in m/s^2^: (**a**) Receiver A; (**b**) Receiver B.

In 2009, the National Geographic Information Institute (NGII) of South Korea conducted airborne gravity surveying for a new geoid model of the Korean peninsula and surrounding marine areas. The survey was flown by Cessna Grand Caravan at speeds of about 280 km/h and a constant altitude of 10,000 feet. GPS data were collected from both GPS receivers and six ground-based continuously operating reference stations (CORS) at an interval of 1 s. GPS measurements were collected using both receivers connected to a single antenna with the kinematic accelerations of the aircraft being computed using the network-based PVA model proposed by [6]. Figure 2 shows the 1.5 h span (~400 km in distance) of 1 htz GPS data collected on 11 January 2009 that were selected for this study. 

**Figure 3:**
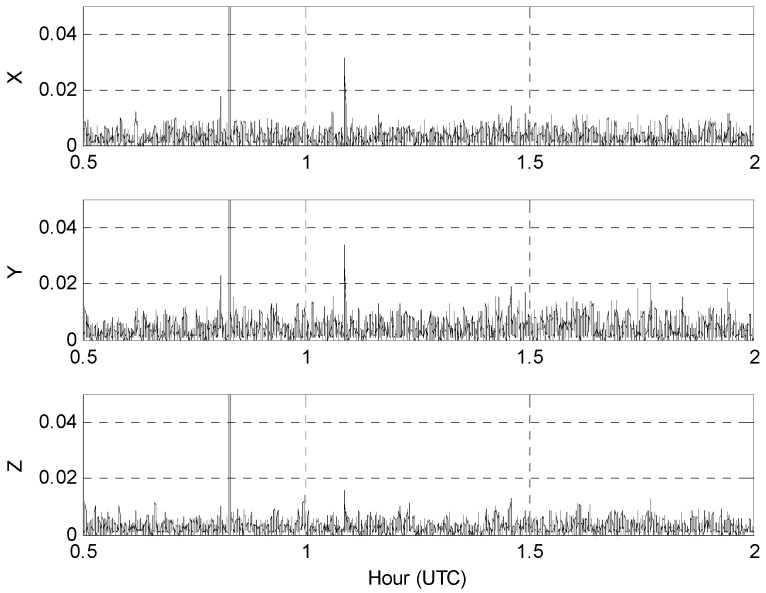
Receiver A minus receiver B acceleration root-mean-squared differences in m/s^2^ from Figure 2.

Figure 3 presents the root-mean-squared (RMS) data differences between the two receivers with the statistical characteristics that are listed in Table 1. 

**Figure 4:**
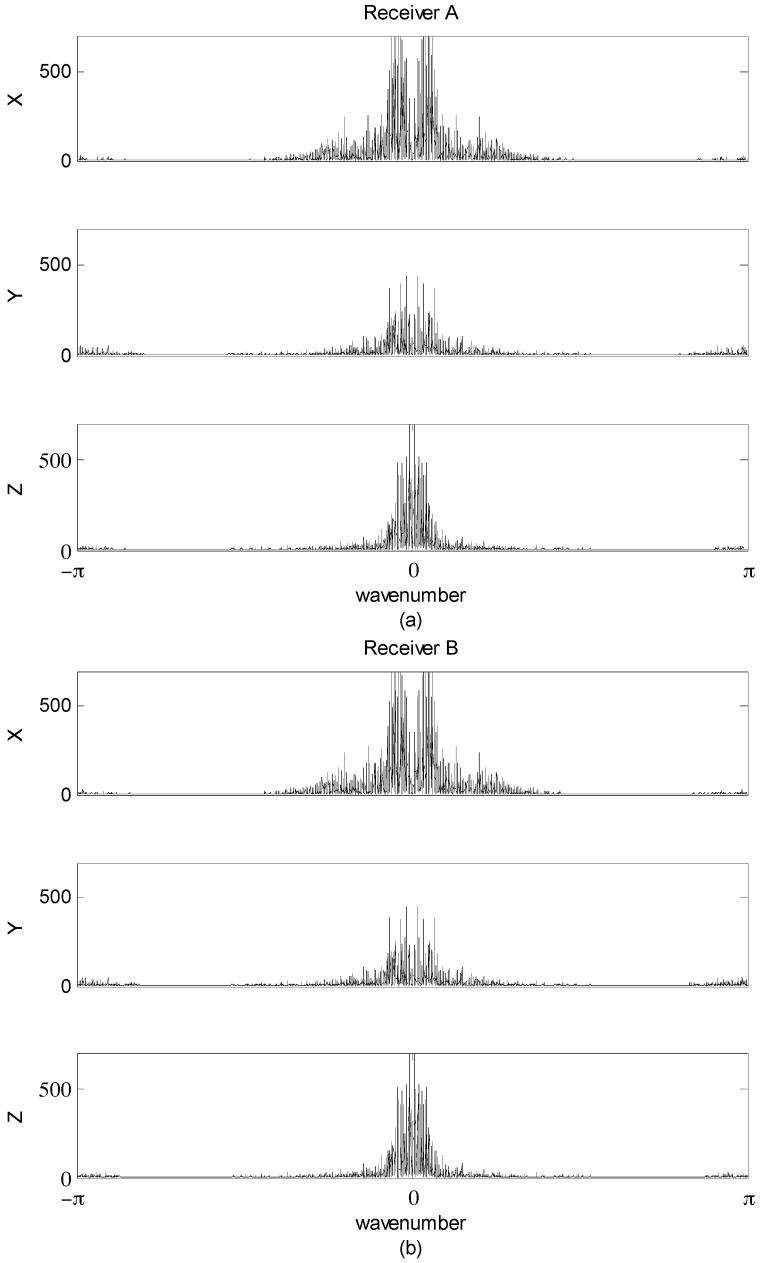
Kinematic acceleration power spectra in m^2^/s^4^: (**a**) Receiver A; (**b**) Receiver B.

The power spectra for the receiver A- and B-measured kinematic accelerations are compared in Figure 4 with similarly dominant patterns of the lower frequency components.

**Figure 5:**
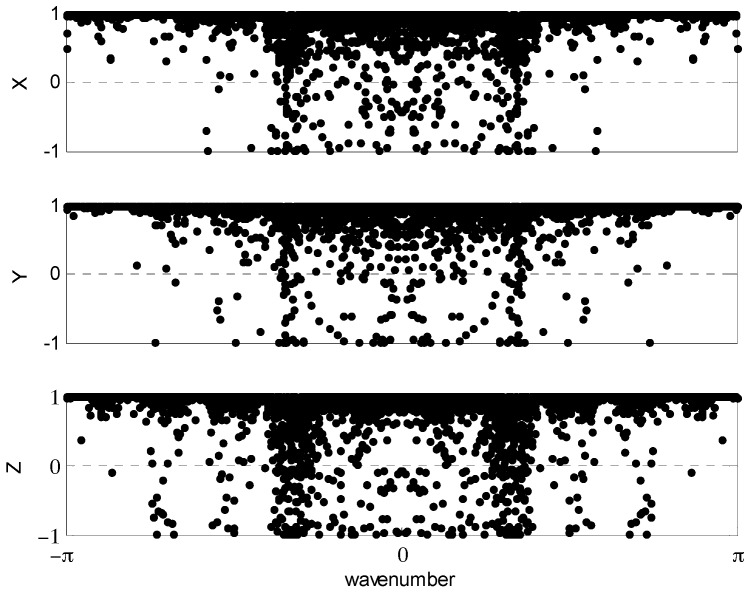
Computed correlation coefficients for each wavenumber.

**Figure 6:**
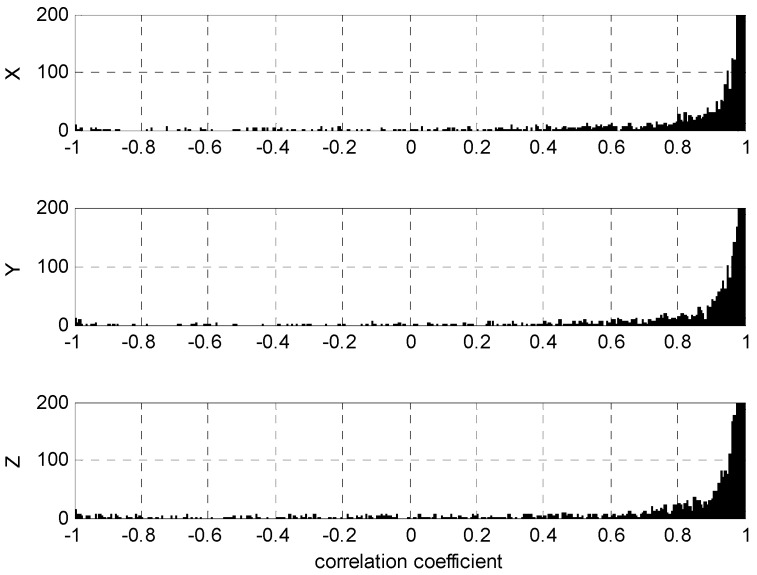
Histograms of correlation coefficients.

The CCs for all wavenumber components pairs were computed by Equation (3) as presented in Figure 5, where relatively high positive correlations are dominant as also reinforced by the histograms of Figure 6. These results suggest that the most robust cutoff or tolerance for constructing the WCF is ε > 0 because negative CCs clearly reflect noise between the two receiver signals from a common antenna. However, the strong gradient change of histograms in Figure 6 indicates that ε ≥ 0.8 or 0.9 may be even more discriminating of the antenna’s signal in both receivers.

**Figure 7:**
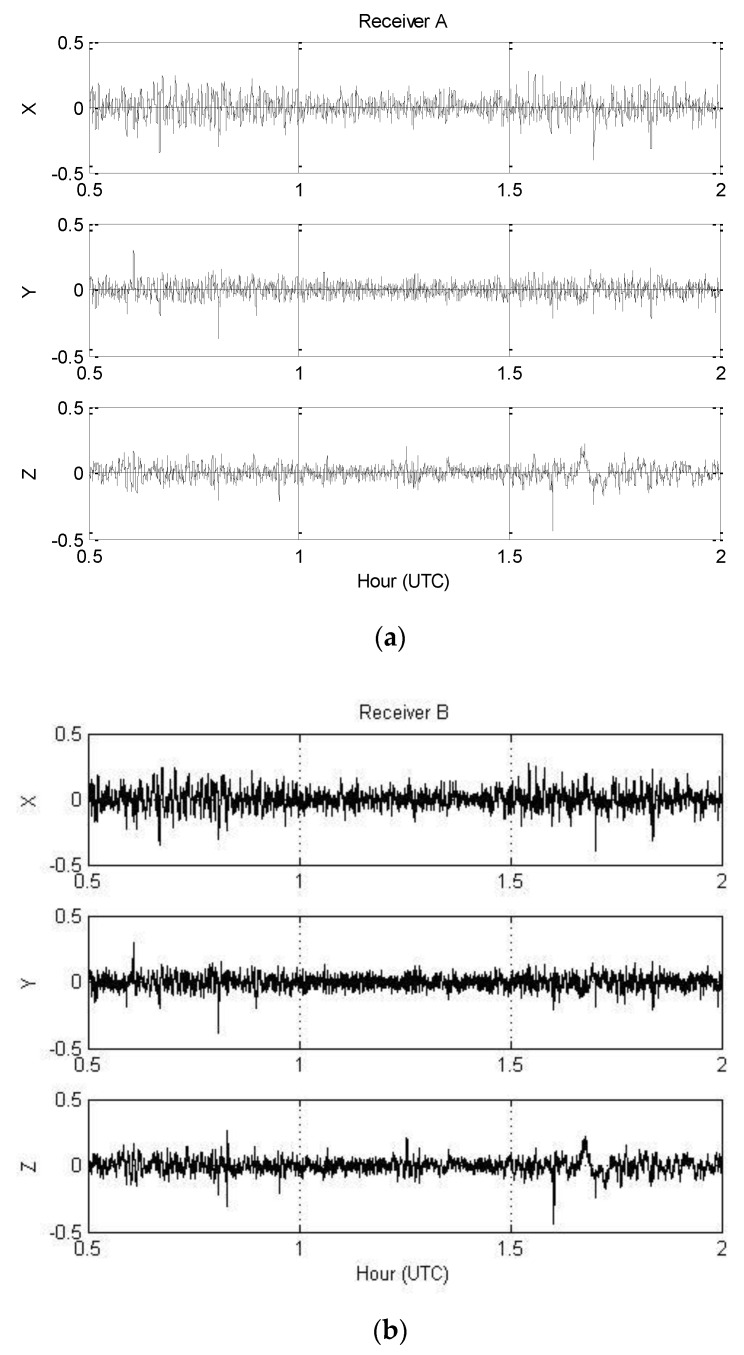
Wavenumber correlation filter (WCF) kinematic accelerations in m/s^2^ using the cutoff ε≥ 0.9: (**a**) Receiver A; (**b**) Receiver B.

Using the tolerance ε≥ 0.9, for example, yields the wavenumber correlation filtered kinematic accelerations presented in Figure 7. 

**Figure 8:**
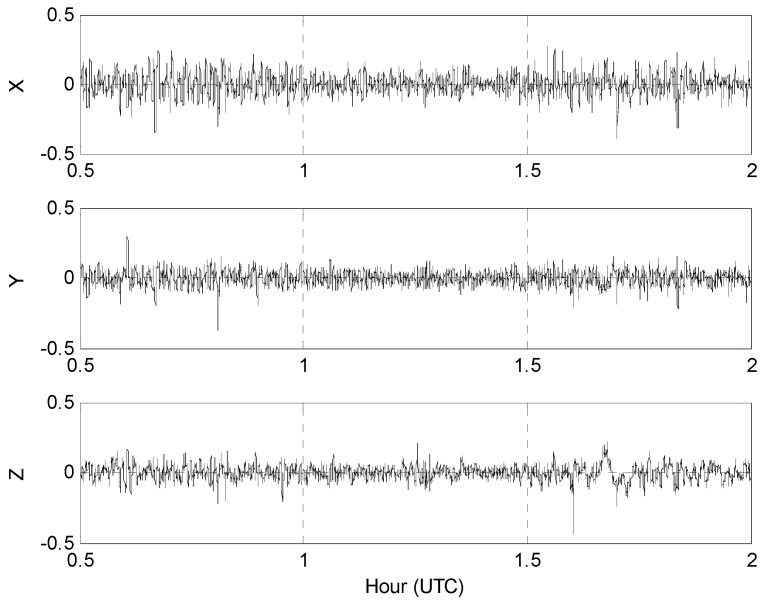
Least squares estimates of the WCF kinematic accelerations in m/s^2^ from 2-point averages of the data in Figure 7 with the root-mean-squared (RMS) errors given in Figure 9.

**Figure 9:**
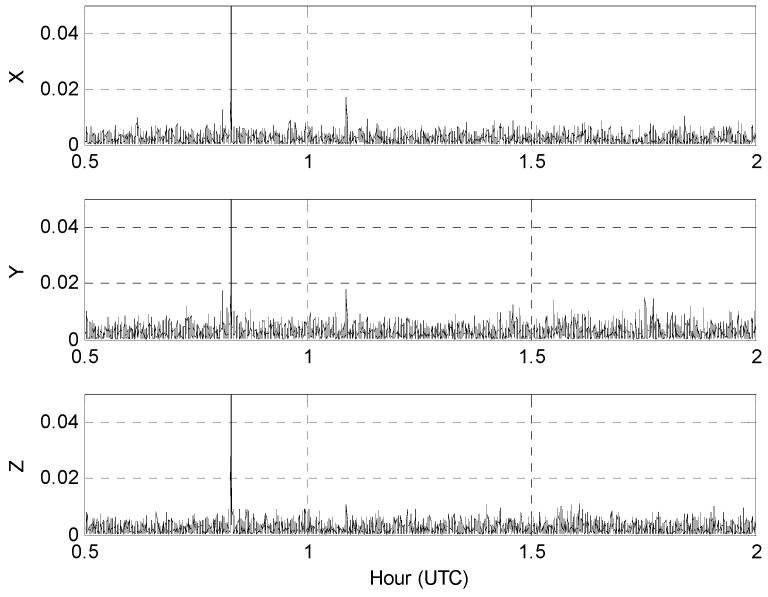
Receiver A minus receiver B WCF acceleration root-mean-squared differences in m/s^2^ from Figure 7.

Figure 8 gives the least squares kinematic acceleration estimates from the 2-point averages of the WCF data in Figure 7 with the RMS errors shown in Figure 9.

Table 2 lists the statistical mean and standard deviation values of the RMS errors along with correlation coefficients and affiliated noise percentages for the WCF kinematic accelerations of Figure 7. 

The Table 1 minus Table 2 differences in mean RMS errors yield reductions of 0.008122 m/s^2^ (812 mGal), 0.0011657 m/s^2^ (116.6 mGal), and 0.0003725 m/s^2^ (37.3 mGal) in the respective X, Y, and Z components. Comparing the noise estimates in the tables also suggests that WCF obtained noise suppression improvements in the X, Y, and Z components of roughly 27%, 27%, and 20%, respectively.

It should be noted, however, that the WCF reduction of noise is but one contributor in the overall error propagation equation of the gravity signal’s estimation. Additional contributions include the effects of the data smoother, end matching of the flight lines, and the coordinate transformation [5]. However, preliminary processing of the raw- and WCF-data with B-splines over 60 s smoothing windows resulted in gravity anomaly estimates with RMS error improvements in mGal of 31.0, 7.1, and 4.7 in the X, Y, and Z components, respectively. These results clearly represent significant improvements in gravity anomaly estimation for the subsurface exploration of the Earth e.g., [13].

## 4. Summary and Conclusions

Effective airborne gravity surveying requires accurate kinematic acceleration determinations. This study investigated the enhancement of the kinematic accelerations determined from GPS data obtained by two receivers from a single antenna. The WCF was applied to extract the positively correlated, larger magnitude (i.e., ε ≥ 0.9) wavenumber components of the kinematic accelerations measured by the two receivers. Combining the WCF data by simple averaging yields least squares estimates of the antenna’s kinematic accelerations with significantly suppressed noise. Thus, WCF of multiple receiver accelerations can be an effective enhancement for airborne gravimetry applications.

## Figures and Tables

**Table 1:** Statistical mean and standard deviation (Std) values of the RMS differences in m/s^2^ between the kinematic accelerations for receivers A and B in Figure 2. The correlation coefficients (CC (A,B)) between the datasets of the two receivers are also listed with the related % noise contributions from Equation (2).

Components	Mean	Std.	CC	% Noise
X	0.0030861	0.0029858	0.9959	6.4
Y	0.0040969	0.0038539	0.9848	12.4
Z	0.0027949	0.0048019	0.9876	11.2

**Table 2:** Statistical mean and standard deviation (Std.) values of the RMS differences in m/s^2^ between the WCF kinematic accelerations for receivers A and B in Figure 7. The correlation coefficients (CC(A,B)) between the datasets of the two receivers is also listed along with the related % noise contributions from Equation (2).

Components	Mean	Std.	CC	% Noise
X	0.0022739	0.0022404	0.99775	4.7
Y	0.0029312	0.0028497	0.99183	9.1
Z	0.0024221	0.0037318	0.99200	9.0
